# The effects of four decades of climate change on the breeding ecology of an avian sentinel species across a 1,500‐km latitudinal gradient are stronger at high latitudes

**DOI:** 10.1002/ece3.7459

**Published:** 2021-03-18

**Authors:** Marta Lomas Vega, Thord Fransson, Cecilia Kullberg

**Affiliations:** ^1^ Department of Zoology Stockholm University Stockholm Sweden; ^2^ Bolin Centre for Climate Research Stockholm University Stockholm Sweden; ^3^ Department of Environmental Research and Monitoring Swedish Museum of Natural History Stockholm Sweden

**Keywords:** adaptation, breeding phenology, latitudinal variation, pied flycatcher, reproductive success, spring advancement

## Abstract

Global warming affects breeding phenology of birds differentially with latitude, but there is contrasting evidence about how the changing climate influences the breeding of migrating songbirds at their northern breeding range. We investigate the effect of climate warming on breeding time and breeding success of European pied flycatchers *Ficedula hypoleuca* in Sweden during a period of 36 years using nest reports from bird ringing. To account for the latitudinal variation, we divided Sweden into three latitudinal bands (northern, intermediate, and southern). We applied a sliding window approach to find the most influential period and environment characteristics (temperature, vegetation greenness, and precipitation), using linear mixed models and model averaging. Our results show a long‐term advancement of breeding time related to increasing spring temperature and vegetation greenness during a period before hatching. Northern breeders revealed a larger advancement over the years (8.3 days) compared with southern breeders (3.6 days). We observed a relatively stronger effect of temperature and greenness on breeding time in the north. Furthermore, northern birds showed an increase in breeding success over time, while birds breeding at southern and intermediate latitudes showed reduced breeding success in years with higher prehatching temperatures. Our findings with stronger environment effects on breeding time advancement in the north suggest that pied flycatchers are more responsive to weather cues at higher latitudes. Breeding time adjustment and, potentially, low competition help explain the higher long‐term success observed in the north. Reduced breeding success at more southerly latitudes suggests an inability to match breeding time to very early and warm springs, a fate that with continued climate change could also be expected for pied flycatchers and other long‐distance migrants at their very northern breeding range.

## Graphical abstract

We investigate the effects of latitudinal variation of climate change on breeding time and reproductive success at the northern breeding range of a long‐distance migratory songbird. Birds breeding at the very north faced a stronger effect on breeding time advancement and their breeding success increased over the years, while more southerly breeding birds faced a lowered breeding success in very warm springs. Our results suggest that there is a risk that migratory birds will be more vulnerable to a continued climate change, especially in the north.

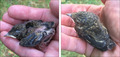

## 1 INTRODUCTION

Evidence of the impact of recent climate change on phenology of animals and plants is ample today (Both et al., 2006; Dunn & Winkler, 1999; Hällfors et al., 2020; Helm et al., 2019; Parmesan & Yohe, 2003; Root et al., 2003; Saino et al., 2011; Schwartz et al., 2006; Shave et al., 2019; Townsend et al., 2013), but the underlying mechanisms of the changing patterns are poorly understood (Knudsen et al., 2011), especially considering the variation of climate change with latitude (Parmesan, 2007). In birds, phenological shifts, like advancement of breeding time due to warmer and earlier springs, may facilitate larger clutches and the opportunity to rear more young (Dunn, 2004). However, phenological changes may also have negative consequences on breeding success and population trends when, for example, sudden changes in weather (Dunn, 2004; Visser et al., 2015) or ecological mismatch occur (Both et al., 2006; Harrington et al., 1999; Saino et al., 2011; Sanz et al., 2003). Thus, in the face of rapid climate change, birds may experience reduced breeding success that can result in population decline (Both et al., 2006; Sanz et al., 2003), but if there is time for adaptive behaviors to emerge, populations could adapt to the novel environments (Charmantier et al., 2008; Helm et al., 2019; Visser et al., 2015). In fact, microevolution on breeding time advancement as a consequence of climate change has been proven in pied flycatchers in Germany (Helm et al., 2019).

Phenological response to climate change has been found to be stronger at higher latitudes due to a more intense effect of global warming toward the north, with temperature increase as the main part of the mechanism (Intergovernmental Panel on Climate Change, IPCC, 2001; Root et al., 2003; Shukla et al., 2019). Still, some studies at northern sites experienced more stable spring and summer temperatures during the last decades (Ram et al., 2019) or even temperature decrease (Both et al., 2004; Both & Marvelde, 2007) finding nonsignificant trends on breeding time change in several bird species. Nevertheless, studies on the effect of climate change on breeding time and breeding success over a large latitudinal gradient are scarce (Parmesan, 2007; but see examples in Dunn & Winkler, 1999; Hällfors et al., 2020; Meller et al., 2018; Shave et al., 2019) and need further investigation.

In seasonal habitats, insectivorous birds generally time their breeding season to coincide with high food availability to match their offspring's needs and thus maximize breeding success (Van Noordwijk et al., 1995). As springs become earlier with climate change, some species have adjusted their breeding time, matching the advancement of local‐insect peak (Both et al., 2006; Charmantier et al., 2008; Emmenegger et al., 2014). In particular, Afro‐Palearctic long‐distance migrants, that fly thousands of km from Tropical Africa, breeding at mid‐latitude forests of Europe may need more generation time than resident birds to adjust breeding to earlier food peaks at breeding areas; to avoid ecological mismatch (Both et al., 2010; Saino et al., 2011; Sanz et al., 2003). Nevertheless, it is unclear whether the underlying mechanism of long‐term avian productivity decrease observed in the UK is directly related to trophic asynchrony (Franks et al., 2017). Furthermore, in boreal forest of Europe, warm spring temperature was positively associated with breeding success suggesting no mismatch between chick food demands and food availability (Meller et al., 2018). Moreover, the tendency to breed early can be triggered by intraspecific competition, rather than trophic synchrony, as suggested for some populations of willow tits (Pakanen et al., 2016) and pied flycatchers (Ahola et al., 2012) in Finland.

The European pied flycatcher has become a model species when studying the effects of climate change on the annual cycle of long‐distance migratory birds (Ahola et al., 2004, 2012; Both et al., 2006; Both & Visser, 2001; Cadahía et al., 2017; Helm et al., 2019; Sanz et al., 2003; Tomotani et al., 2018; Visser et al., 2015). In general, its breeding time has advanced during the last decades in central and western Europe, presumably driven by an increase in spring temperature (Both et al., 2004; Both & Visser, 2001; Helm et al., 2019; Tomotani et al., 2018). At more northern sites, there are reports of less of a change in breeding time (Ahola et al., 2004; Both et al., 2004; Källander et al., 2017; Ram et al., 2019), but a recent study showed earlier date of egg laying over the last three decades that closely associated with birch leafing and higher May temperature (Järvinen, 2020). In southern and central Europe, several populations of pied flycatchers that did not advance their breeding time despite the increase in spring temperatures had lower breeding success (Both et al., 2006; Sanz et al., 2003), while populations that adapted to earlier breeding did not decline (Both et al., 2006). However, early breeding can cause lower adult survival during possible subsequent cold springs, especially after long‐distance migration (Visser et al., 2015) or due to emergent competition effects (Ahola et al., 2007; Samplonius & Both, 2019). Nevertheless, contrasting results are typically assigned to different rates of climate change between latitudes and study periods (Parmesan, 2007; Visser et al., 2015), and there are also population and habitat‐specific differences (Vatka et al., 2011). In addition, differences in the methods used may add to the variability of results observed (Parmesan, 2007).

Temperature has been widely used as a single variable representing climate, as it strongly correlates with spring phenology changes (Both et al., 2006; Harrington et al., 1999). Certainly, research has shown that birds can breed earlier as a response to increasing spring temperatures (Both et al., 2004; Crick et al., 1997; Dunn, 2004; Dunn & Winkler, 1999; Källander et al., 2017; Kluen et al., 2017; McDermott & DeGroote, 2016; Parmesan & Yohe, 2003; Ram et al., 2019; Samplonius et al., 2018; Shave et al., 2019; Townsend et al., 2013). However, increased spring precipitation may also affect reproduction, especially at relatively low temperatures, contributing to later breeding time and lower breeding success (Cox et al., 2020; Dunn, 2004; McDermott & DeGroote, 2016). Furthermore, primary productivity may influence breeding through its effect on habitat quality (Gordo, 2007). Although the combination of warm spring temperature and high precipitation will trigger primary productivity, measurements of seasonal vegetation greenness give a more direct information on vegetation development, in a given space and time (Gordo, 2007; Pettorelli et al., 2005). Moreover, several studies indicate association between vegetation greenness and spring phenology advancement as well as insectivore food resources (Fernández‐Tizón et al., 2020; Jørgensen et al., 2016; Thorup et al., 2017; Vega & Thorup, 2018; but see Rybinski et al., 2016; Veen et al., 2010). Altogether, a set of environmental variables such as temperature, precipitation, and vegetation greenness can be used to disentangle the mechanism of climate change effects on breeding phenology and reproduction.

Here, we investigate the influence of spring advancement on timing of breeding and reproductive success of the pied flycatcher breeding in Sweden during a period of 36 years. Our study is unique in covering a large span of latitudes (the whole range of Sweden with more than 1,500 km) and over a long time span, allowing us to investigate differences in the effects of climate change depending on latitude in the northern breeding range of small songbirds (but see other studies covering latitudinal gradients: Dunn & Winkler, 1999 and Shave et al., 2019 for North America, and Meller et al., 2018 and Hällfors et al., 2020 for Finland). Sweden has since the 1980s experienced higher year‐round temperature and earlier and longer vegetation periods, especially in the north where the vegetation period lengthened by two weeks during 40 years (Swedish Meteorological and Hydrological Institute, SMHI, 2021b, 2021d). Being a migratory bird, breeding time and success of pied flycatchers might be affected by environmental factors during migration, but we chose to restrict our analyses to study effects of environmental conditions at the breeding ground. We used standardized nest reports collected all over Sweden with information on ringing date of chicks, number of chicks alive, and developmental stage of the chicks per nest. As environmental variables, we used temperature, precipitation and a remote‐sensing measurement of primary productivity, the normalized difference vegetation index (NDVI), measured all over Sweden, giving us the opportunity to better understand the geographical differences in breeding response to climate change found to date (Both & Marvelde, 2007; Källander et al., 2017; Ram et al., 2019; Samplonius et al., 2018), and shed light on the mixed results found for Scandinavia. The potential of NDVI use in this kind of studies has been generally overlooked. To investigate the mechanism of breeding response to global warming at a regional scale, we use NDVI as an estimate of seasonal vegetation development (and consequently spring advancement; see, e.g., Emmenegger et al., 2016; Jørgensen et al., 2016; Kristensen et al., 2013; Thorup et al., 2017 for studies on a more global scale). We quantified the relationship between the environmental variables and breeding time, and the effect of these on reproductive success at different latitudes over Sweden. Within a model‐averaging framework and sliding window approach, we identify important variables and period of the year influencing breeding time and success.

We predict earlier breeding time in relation to increasing spring temperatures and vegetation greenness throughout the study period since warmer conditions and higher vegetation greenness (the latter influenced by more precipitation early in spring) enable an earlier onset of breeding. However, we hypothesize that more precipitation near the breeding season could delay breeding time since it would trigger lower temperature and insect availability in a short term. Furthermore, we test whether breeding success of pied flycatchers has been retained, suggesting a sufficient adjustment of breeding time in relation to climate change. In our analysis, we test the hypothesis of the effect of the changing climate in a long term on breeding time and success with the interaction between year and the three environmental variables respectively, thus giving information on the magnitude of climate change effect over time. In addition, we analyze the individual effect of the environment variables to grasp the effects due to their yearly variation, thus regardless of the time trend. In terms of latitudinal variation of climate change, we predict stronger response to climate change toward northern Sweden where, overall, the rate of climate change has been larger. Since spring arrival is later at higher latitudes, we accounted for latitudinal effects in our analyses to avoid confounding effects on the ecological responses to climate change. In the same way, we took into account the possible effect of the sea, given that spring phenology is generally earlier at the coast, by using a distance to the coast variable.

## 2 METHODS

### 2.1 Data collection and processing

**FIGURE 1:**
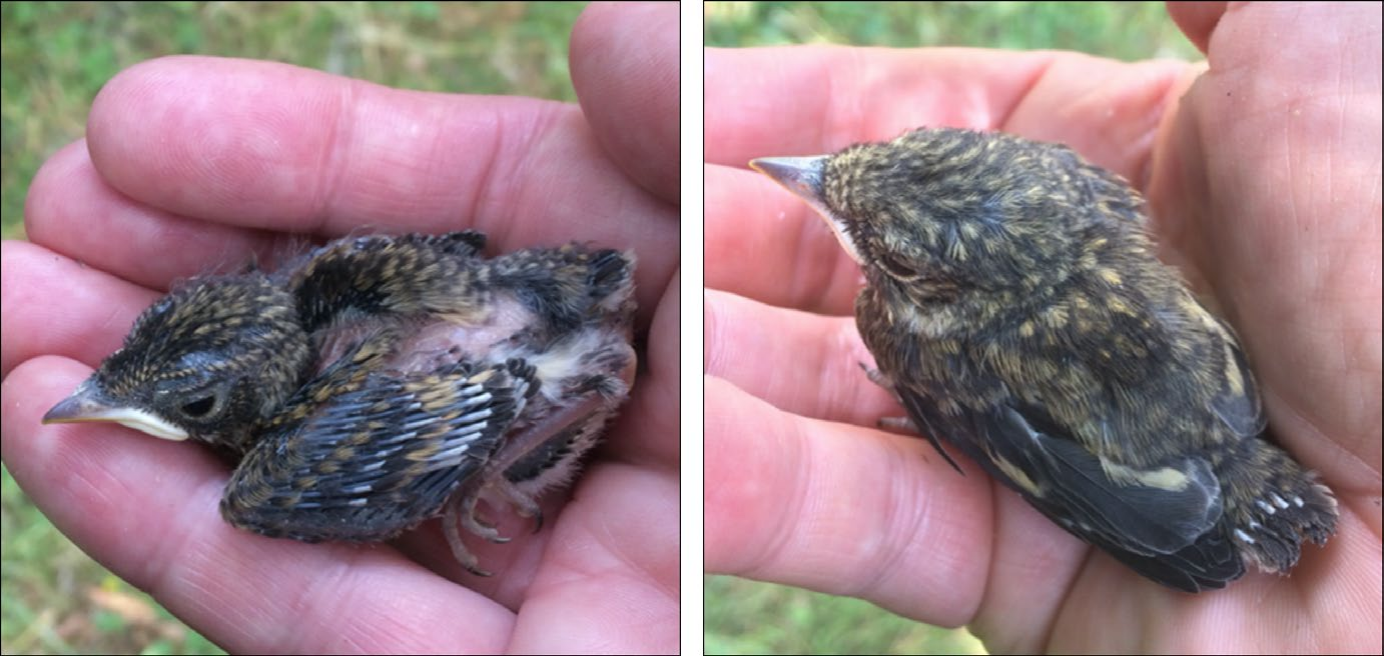
Pied flycatcher nestlings at 8 (left) and 11 (right) days old with developmental stages C and D, respectively

Volunteer ringers licensed by the Swedish Museum of Natural History collect data on ringed nestlings in nest boxes throughout Sweden. We analyzed all available digitized nest reports of pied flycatchers during a 36‐year period spanning from 1982–2017 (no available data during two periods: 1989–1992, 2000–2005; total number of nest reports: 38,050). Each nest report contained information on ringing date, location, chick developmental stage (A, B, C, D, E and X; Table 1), and number of alive chicks. To investigate the change in breeding time, we estimated the hatching day‐of‐year for each clutch using its corresponding chick developmental stage with a standardized description (instructions to ringers from the Museum; Table 1) and the ringing date of nestlings. We established mean age in days for each developmental stage using pictures of pied flycatcher nestlings of known age, taken at a nest box at Tovetorp Research Station, in south of Sweden in 2018 (e.g., in Figure 1). We calculated the hatching day‐of‐year of each clutch by subtracting the mean age in days for their developmental stage from the ringing date (Table 1). To study breeding success, we used the number of ringed chicks per nest and considered this a surrogate of breeding success up to ringing time. We did not use data with stage E (newly fledged, 100 nests) since we wanted to record breeding success (as number of ringed chicks in the nest), or nest data with code X (mixed stages in the same nest, 270 nests).

**TABLE 1:** Chick developmental stages used to calculate age of nestlings, and corresponding age range derived from pictures of nestlings of known age (e.g., in Figure 1)

Chick developmental stage	Description (referred to primary feathers)	Days‐of‐age range	Mean age in days
A	Pin feathers hardly visible	0–3	1.5
B	Visible pin feathers but no visible vanes (only rachis)	4–7	5.5
C	At least one pin feather with visible vane, that covers less than half of the feather's length	8–10	9
D	At least one pin feather with visible vane, that covers more than half of the feather's length	11–15 (16)	13
E	Newly fledged, close to nest	NA	
X	Mixed developmental stages in same nest	NA	

Note

Mean age in days was calculated from the days‐of‐age range.

**FIGURE 2:**
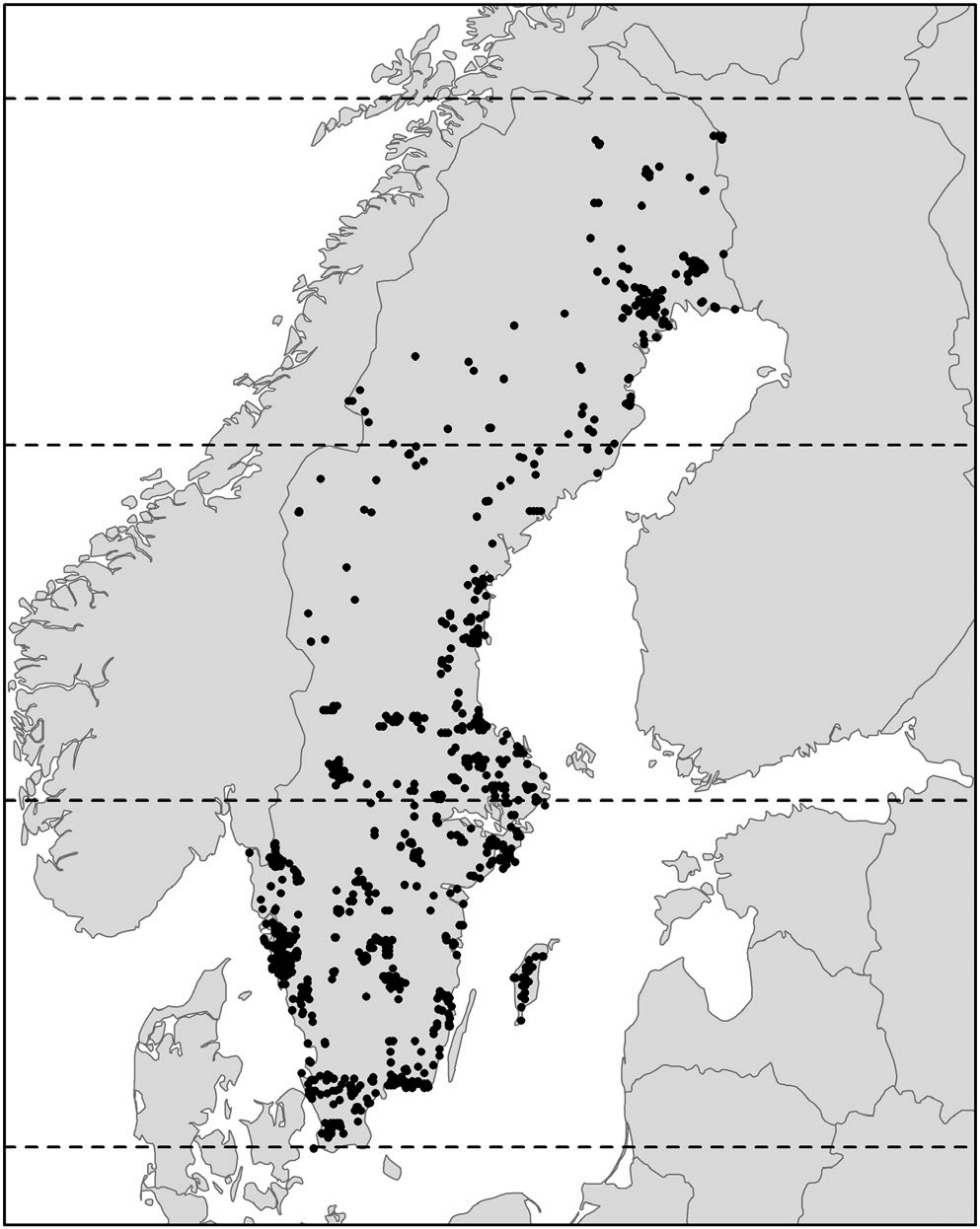
Study area divided into southern (55.4°N–59.7°N), intermediate (59.7°N–64.1°N), and northern (64.1°N–68.4°N) latitudinal bands with locations of nest boxes analyzed (black dots; *N* = 1,077) from 1982–2017

Environmental data consisting of weather and vegetation greenness for the study period were included in the analyses as proxies of climate since these factors closely vary with climate change (IPCC, 2001; Pettorelli et al., 2005), and biological systems generally have capacity to adjust to environmental conditions (Root et al., 2003; Saino et al., 2011). Regional reanalysis weather data were collected from the Copernicus Climate Change Service (https://cds.climate.copernicus.eu/cdsapp#!/dataset/reanalysis‐uerra‐europe‐single‐levels?tab=overview) that provides modeled data of weather observations from the SMHI and statistical simulations. As weather variables, we used surface‐air average and minimum temperature at 2‐m height above the surface and total precipitation, at a spatial resolution of 121 km^2^ (UERRA‐HARMONIE system). As a measurement of primary productivity, we used NDVI from Global Inventory Modeling and Mapping Studies (GIMMS; NDVI3g version 1.1; Pinzon & Tucker, 2014) at a spatial resolution of 85.6 km^2^. NDVI is a remote‐sensing measurement that indicates levels of photosynthetic activity, and it is frequently represented in a scale between 0 and 1, with higher values corresponding to increasing vegetation greenness. NDVI is widely used as a measure of vegetation phenology and indirect indication of food resources that vary with climate conditions (Pettorelli et al., 2005; Sanz et al., 2003; Thorup et al., 2017). It is usual to find a small fraction of not available NDVI data because of atmospheric conditions (e.g., cloud cover and aerosols) even though efforts are dedicated to minimize this (Pinzon & Tucker, 2014). Since the smallest available resolution for the GIMMS NDVI data is biweekly, we also used this temporal resolution for temperature and precipitation. The geographical location of the nest boxes was given by a variable named ‘location ID’ that contained the same longitude and latitude coordinates for several nest boxes, and there were 1,077 location IDs in the final data set (Figure 2). We obtained the environment data from netCDF files using the R package *ncdf4* and extracted values at the corresponding nest location ID and biweekly periods using the R package *raster*. After filtering out NDVI and chick developmental stage NAs (around 1,500 and 7,370 data points, respectively), the final data set contained 29,035 nest reports.

We controlled for the latitude effect in two ways: dividing Sweden into three latitudinal bands and accounting for the effect of latitude as a continuous variable within these areas. The rationale for analyzing each band separately was the uneven distribution of nests across latitudes (Figure 2; southern: 55.4°N–59.7°N, *N* = 17,687; intermediate: 59.7°N–64.1°N, *N* = 5,208; and northern: 64.1°N–68.4°N, *N* = 6,140). In an exploratory analysis, where we analyzed data for all latitudes (with a similar design as for the final analysis within bands, including the use of latitude as a continuous variable), the main effects in the south overshadowed effects only prominent in the north of Sweden. Thus, we considered that an analysis by latitudinal bands was more informative to elucidate different effects over Sweden. We used latitude as a continuous variable within the latitudinal band analysis to control more directly for the fact that breeding time is generally earlier toward the south because of earlier spring phenology, and, consequently, breeding success would be affected. Distance to the (nearest) coast was calculated for all nest locations using a shapefile of coastlines of Sweden. Due to mixed effects linked to topography (e.g., mountains in the north and the two coastlines in the south), we transformed this variable into a factor with three levels fitting our analysis with a general measure of the influence of the sea. The three levels were the closest to the coast (level 1), the intermediate (level 2), and the most inland (level 3). For the analysis at the southernmost latitudinal band, a distance to the coast factor with two levels substituted the three‐level factor given the small amount of data points at level 3.

### 2.2 Statistical analysis

**FIGURE 3:**
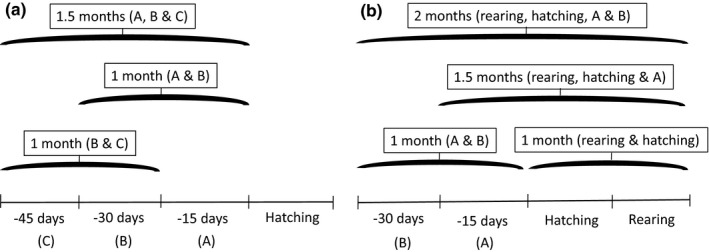
Periods used with sliding window approach to study environmental effects on breeding time (a) and breeding success (b). For breeding time, the environment data used in the analysis were calculated from the average environment values of three periods before hatching time, and for breeding success, we used four periods before hatching and during hatching and rearing (in boxes). Each section in the axes (A, B, C, hatching and rearing) is a 15‐day period in relation to the mean hatching period per latitudinal band (mean hatching occurred first half of June at southern and intermediate latitudinal bands and second half of June at northern band)

To study the effect of climate change on breeding time and breeding success throughout the study period, we used the individual nest data to do model‐averaging analyses with linear mixed‐effect models. We also calculated the change in annual‐average hatching day‐of‐year, breeding success, and seasonal environmental conditions over time with linear regressions to understand their general trends. Estimated hatching day‐of‐year and number of ringed chicks per nest were used as response variables, respectively. Average environment data at time windows before and after (only for breeding success) hatching were used to evaluate both the effect of the environment and which period influenced breeding time and breeding success the most (‘sliding window’ approach, Husby et al., 2010). For the selection of periods with environmental data to analyze, we calculated the mean hatching time per latitudinal band and obtained, as a reference, the biweekly periods corresponding to these mean hatching times. These mean hatching periods were first half of June in the southern and intermediate latitudinal bands and second half of June in the northern band. Using environmental values relative to these mean hatching periods, we simulated how birds adjusted to climate change in different periods before the reference mean hatching time for the latitudinal bands. In this way, we focus on investigating long‐term seasonal effects and not intraseasonal weather effects. When analyzing the effect of environmental factors on breeding time, three time windows before hatching were used, with environmental means of the three periods (1 or 1.5 months long; Figure 3a). When analyzing the effect of environmental factors on breeding success, we used environmental means of four periods, with periods at hatching plus rearing and before hatching (1, 1.5, or 2 months long; Figure 3b). Each time window was represented with an initial model. Model averaging was run for each response variable per latitudinal band and assessing the influence of the environment on breeding time and breeding success at the corresponding time windows.

Initial models included the interaction terms year * temperature, year * NDVI, and year * precipitation to account for the effect of the changing environment through the years, and for breeding time analysis, there were three models per latitudinal band (each one using the corresponding time window). The interaction terms were not highly correlated (Pearson's coefficients between −0.36 and +0.47; Dormann et al., 2013), and the positive correlations were only between year * temperature and year * NDVI indicating that higher temperature over time associates with higher NDVI, and more precipitation associates with lower temperature and lower NDVI during the same time windows in our study area. We included fixed effects of latitude and distance to the coast as continuous and categorical variables, respectively. When analyzing breeding success, we included hatching day‐of‐year as fixed effect—to account for the intraseasonal effect of breeding time—and chick age since it could also have effect on our measurement of breeding success. The number of initial models to study the variation of breeding success was four per latitudinal band (corresponding to the four time windows used in breeding success analysis). To control for spatial autocorrelation, we included the location ID of the nests as a random factor (*N* = 1,077) in all models. All continuous variables were standardized to mean = 0 and *SD* = 1.

Our criterion for model selection followed an information theoretic approach (Grueber et al., 2011) using the Akaike information criterion (AIC). The best‐supported models had the lowest AIC value or it differed ≤2 units from the top‐competing model (AICs are then considered equivalent; Burnham & Anderson, 2002). Top‐competing models are shown in Supporting Information (Tables S1 and S2). Akaike weights (relative importance), confidence intervals, and coefficients of model‐averaged parameters were calculated per latitudinal band. We used the environment at the most influential periods to show the annual‐average trends.

We calculated Moran's Index on the best‐model residuals confirming that there was no spatial autocorrelation (*p* ≥ .05). Collinearity between independent variables was assessed with Pearson's coefficients (threshold ≤0.7), variance inflation factor for breeding time analysis (VIF; threshold ≤2.5), and using univariate model comparison for breeding success analysis (Dormann et al., 2013). The pairwise correlation of all our model terms within a particular model was below the mentioned correlation thresholds; thus, we considered that there were not highly correlated. Models with minimum temperature were not used in the final analysis since they were less supported than models using mean temperature. We used the R packages *lme4* for mixed‐effect models and *MuMIn* for model averaging. All statistical analyses were carried out in R 3.5.2 64‐bit (R Core Team, 2019).

## 3 RESULTS

### 3.1 Environmental parameters

At the southern and intermediate latitudinal bands, environmental means during May (average of 30 and 15 days before mean hatching time; Figure 3) had a higher impact on the breeding time and breeding success than the other periods analyzed (averaged models with the environment during May had lower AIC; Table 2 and Table S1). At the northern latitudinal band, environmental means from May to first half of June had more effect on breeding time (Table 2 and Table S1). However, in the best models of the northern band, the environment at the periods analyzed did not explain variation of breeding success (Table 3 and Table S2). Thus, the most influential period was from May until mean hatching time for the three latitudinal bands. We will from here on call this period ‘pre‐hatching period’ and refer to the environmental conditions during this period as prehatching temperature/NDVI/precipitation.

**TABLE 2:** Summary of results of breeding time

Breeding time					
Parameters	Estimate	*SE*	CI 2.5%	CI 97.5%	*w_i_*
Southern 55.4°N–59.7°N					
Intercept	−0.02	0.04	−0.11	0.06	
Latitude	0.31	0.06	0.19	0.43	1.00
Temperature A & B	−0.34	0.01	−0.36	−0.32	1.00
Year	−0.10	0.01	−0.12	−0.07	1.00
NDVI A & B * Year	−0.09	0.01	−0.11	−0.07	1.00
Temperature A & B * Year	0.06	0.01	0.04	0.09	1.00
NDVI A & B	−0.01	0.01	−0.03	0.01	1.00
Intermediate 59.7°N–64.1°N					
Intercept	−0.17	0.07	−0.30	−0.04	
Latitude	0.37	0.13	0.11	0.63	0.96
Temperature A & B	−0.28	0.02	−0.31	−0.24	1.00
Year	−0.10	0.04	−0.18	−0.02	1.00
NDVI A & B * Year	−0.10	0.02	−0.13	−0.06	1.00
Rain A & B	0.07	0.01	0.04	0.09	1.00
Distance to the coast (2‐1)^a^	−0.14	0.10	−0.34	0.07	0.96
Distance to the coast (3‐1)^a^	0.33	0.12	0.10	0.56	0.96
Distance to the coast (3‐2)^a^	0.47	0.15	0.17	0.76	0.96
NDVI A & B	0.02	0.02	−0.02	0.06	1.00
Temperature A & B * Year	0.06	0.03	0.00	0.12	0.84
Northern 64.1°N–68.4°N					
Intercept	1.83	0.24	1.35	2.31	
Latitude	−0.72	0.14	−0.99	−0.45	1.00
Temperature A, B & C	−0.43	0.02	−0.47	−0.38	1.00
Year	−0.04	0.03	−0.10	0.01	1.00
Temperature A, B & C * Year	0.15	0.02	0.11	0.18	1.00
NDVI A, B & C	−0.08	0.02	−0.11	−0.05	1.00
Rain A, B & C	0.01	0.02	−0.03	0.06	0.26
Distance to the coast (2‐1)^a^	0.06	0.13	−0.19	0.32	0.25
Distance to the coast (3‐1)^a^	0.05	0.11	−0.16	0.27	0.25
Distance to the coast (3‐2)^a^	−0.01	0.08	−0.16	0.14	0.25

Note

Model‐averaged parameters with estimates, standard errors (*SE*), confidence intervals (CI), and Akaike weights (*w_i_*). Results per latitudinal band where the period from May until mean breeding time (the so‐called prehatching period) was more supported based on lowest AIC of competing models. A, B & C refer to 15‐day periods in relation to mean hatching period as in Figure 3. ‘Full’ average method was used. Parameters with *w_i_* < 0.2 are not included.

^a^ Distance to the coast compares relative effects in relation to the variable levels; for example, distance to the coast (2‐1) shows the effect of level 2 compared with level 1.

**TABLE 3:** Summary of results of breeding success

Breeding success					
Parameters	Estimate	*SE*	CI 2.5%	CI 97.5%	*w_i_*
Southern 55.4°N–59.7°N					
Intercept	−0.05	0.04	−0.12	0.03	
Hatching day‐of‐year	−0.38	0.01	−0.40	−0.36	1.00
Chick age	−0.15	0.01	−0.17	−0.13	1.00
Temperature A & B	−0.06	0.01	−0.09	−0.03	1.00
Latitude	0.13	0.06	0.01	0.24	0.89
Intermediate 59.7°N–64.1°N					
Intercept	−0.06	0.03	−0.11	−0.01	
Hatching day‐of‐year	−0.28	0.02	−0.32	−0.25	1.00
Chick age	−0.09	0.02	−0.13	−0.06	1.00
Temperature A & B	−0.12	0.02	−0.15	−0.09	1.00
Northern 64.1°N–68.4°N					
Intercept	0.35	0.07	0.22	0.49	
Hatching day‐of‐year	−0.34	0.02	−0.37	−0.31	1.00
Chick age	−0.14	0.02	−0.17	−0.10	1.00

Note

Model‐averaged parameters with estimates, standard errors (*SE*), confidence intervals (CI), and Akaike weights (*w_i_*). Results per latitudinal band where the period from May until mean breeding time (the so‐called prehatching period) was more supported based on lowest AIC of competing models. A & B refer to 15‐day periods in relation to mean hatching period as in Figure 3. ‘Full’ average method was used. Parameters with *w_i_* < 0.2 are not included.

**FIGURE 4:**
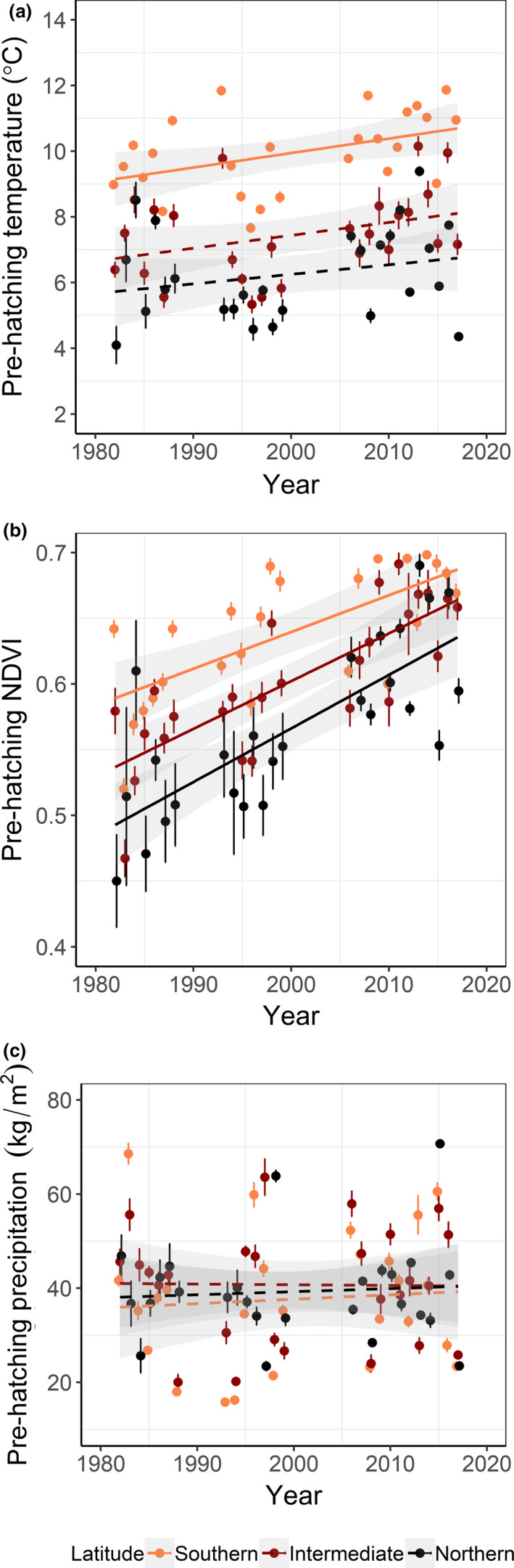
Prehatching environment (from May until mean breeding time), measured as annual averages of temperature (a), vegetation greenness (NDVI; b), and precipitation (c), for pied flycatchers breeding in Sweden from 1982–2017, split by three latitudinal bands. Linear regressions with *p* ≤ .05 (solid lines) and *p* > .05 (dashed lines), standard errors (bars), and confidence intervals (shaded) are shown

At the southern latitudinal band, pied flycatchers experienced a significant average prehatching temperature increase of 1.44°C over the years (+0.04°C/year, *N* = 26 years, *b* (slope) = 0.04, *SE* = 0.02, *t*_24_ = 2.37, adjusted *R* ^2^ = 0.16, *p* = .03; Figure 4a). At the intermediate and northern bands, the average prehatching temperature did not significantly change (intermediate, *b* = 0.04, *SE* = 0.02, *t*_24_ = 1.82, adjusted *R* ^2^ = 0.08, *p* = .08; northern, *b* = 0.03, *SE* = 0.02, *t*_24_ = 1.23, adjusted *R* ^2^ = 0.02, *p* = .23; Figure 4a). Average prehatching NDVI increased 0.1 (scale 0–1) during the study period across latitudinal bands (southern, *b* = 0.003, *SE* = 0.001, *t*_24_ = 4.86, adjusted *R* ^2^ = 0.47, *p* < .001; intermediate, *b* = 0.004, *SE* = 0.001, *t*_24_ = 6.34, adjusted *R* ^2^ = 0.61, *p* < .001, and northern, *b* = 0.004, *SE* = 0.001, *t*_24_ = 5.77, adjusted *R* ^2^ = 0.56, *p* < .001; Figure 4b). Pied flycatchers did not experience any directional change in prehatching precipitation over the years (southern, *b* = 0.09, *SE* = 0.24, *t*_24_ = 0.38, adjusted *R* ^2^ = −0.03, *p* = .71; intermediate, *b* = −0.01, *SE* = 0.21, *t*_24_ = −0.07, adjusted *R* ^2^ = −0.04, *p* = .94, and northern, *b* = 0.06, *SE* = 0.18, *t*_24_ = 0.36, adjusted *R* ^2^ = −0.04, *p* = .72; Figure 4c).

### 3.2 Effects on breeding time

**FIGURE 5:**
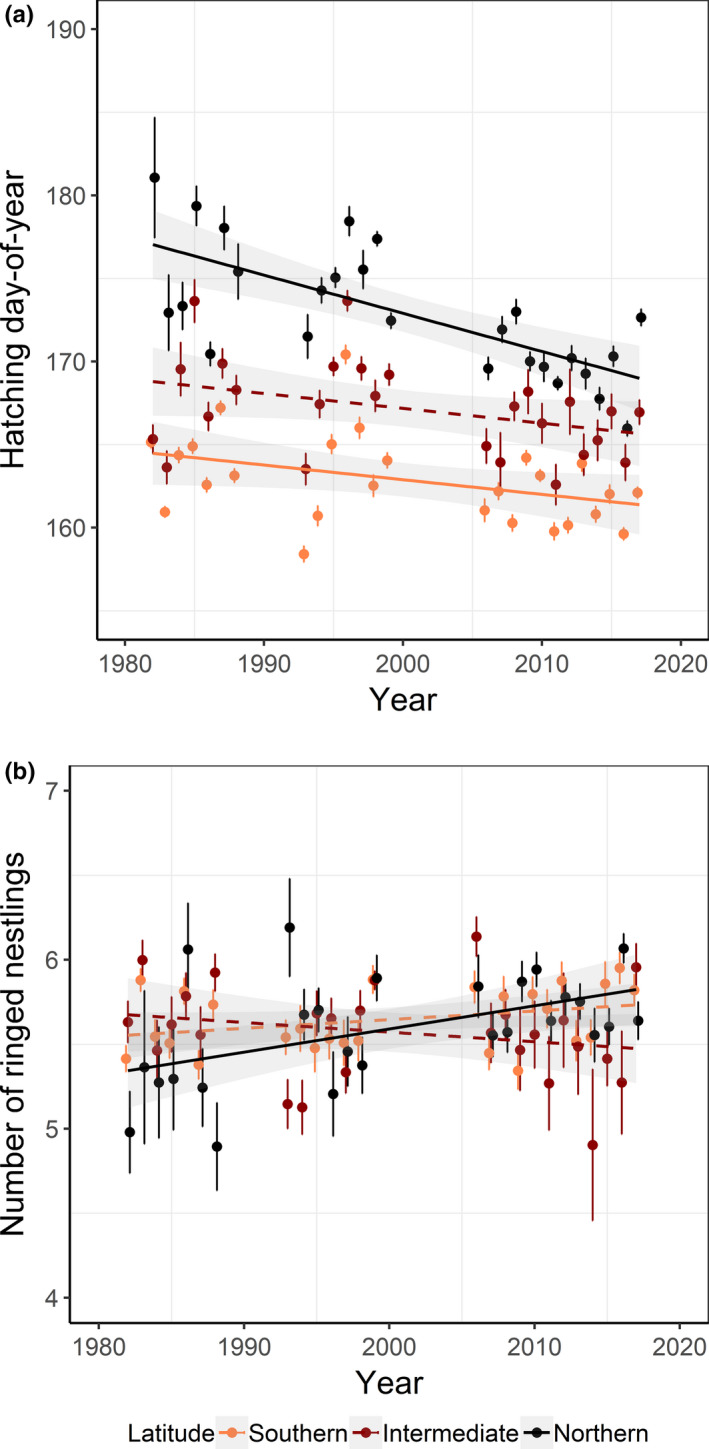
Breeding time (a) and breeding success (b), measured as hatching day‐of‐year and number of ringed nestlings, respectively. Annual averages for pied flycatchers breeding in Sweden during 1982–2017 split by three latitudinal bands. Linear regressions with *p* ≤ .05 (solid lines) and *p* > .05 (dashed lines), standard errors (bars), and confidence intervals (shaded) are shown

**FIGURE 6:**
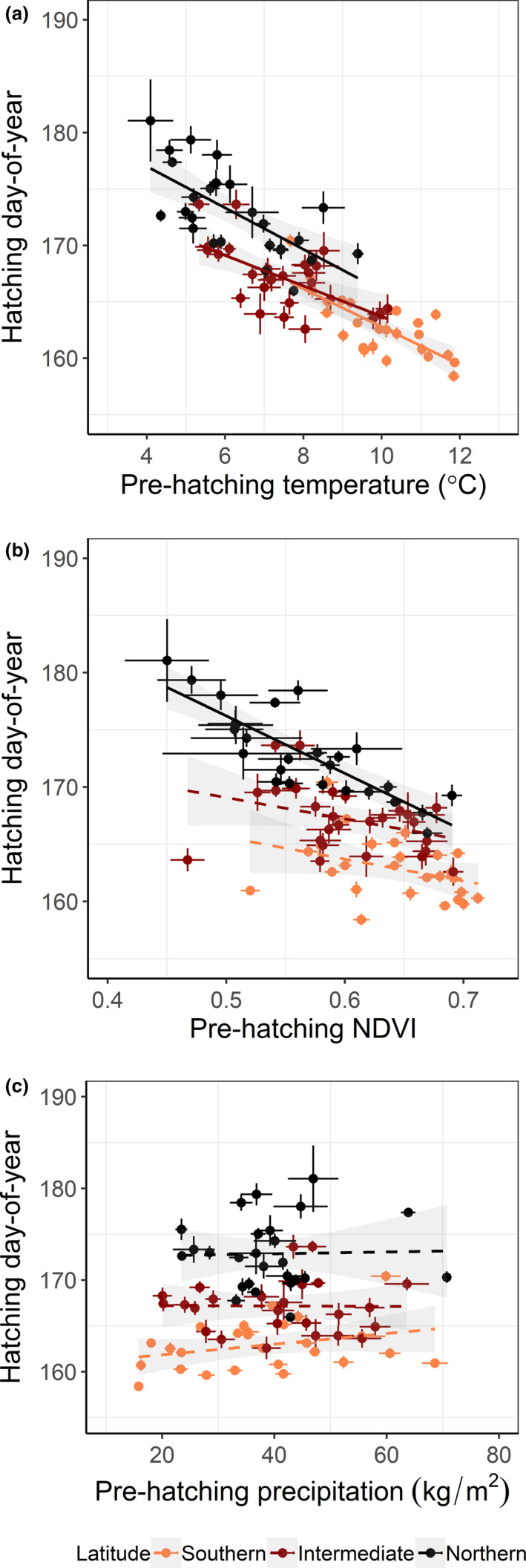
Breeding time, measured as hatching day‐of‐year, in relation to prehatching environment (from May until mean breeding time) using temperature (a), vegetation greenness (b), and precipitation (c), Annual averages for pied flycatchers breeding in Sweden from 1982 to 2017 split by three latitudinal bands. Linear regression of breeding time ~ temperature, *N* = 26 years: southern, *b* (slope) = −1.68, *SE* = 0.30, *t*_24_ = −5.58, adjusted *R* ^2^ = 0.55, *p* < .001; intermediate, *b* = −1.35, *SE* = 0.34, *t*_24_ = −3.95, adjusted *R* ^2^ = 0.37, *p* < .001, and northern, *b* = −1.83, *SE* = 0.40, *t*_24_ = −4.53, adjusted *R* ^2^ = 0.44, *p* < .001. Linear regression of breeding time ~ NDVI (scale 0–1): southern, *b* = −19.15, *SE* = 1.01, *t*_24_ = −1.89, adjusted *R* ^2^ = 0.09, *p* = .07; intermediate *b* = −18.59, *SE* = 1.01, *t*_24_ = −1.84, adjusted *R* ^2^ = 0.09, *p* = .08, and northern, *b* = −50.16, *SE* = 0.69, *t*_24_ = −7.26, adjusted *R* ^2^ = 0.67, *p* < .001. Linear regression of breeding time ~ precipitation: southern, *b* = 0.06, *SE* = 0.04, *t*_24_ = 1.58, adjusted *R* ^2^ = 0.06, *p* = .13; intermediate band: *b* = −0.002, *SE* = 0.05, *t*_24_ = −0.05, adjusted *R* ^2^ = −0.04, *p* = .96, and northern, *b* = 0.01, *SE* = 0.07, *t*_24_ = 0.13, adjusted *R* ^2^ = −0.04, *p* = .89. Linear regressions with *p* ≤ .05 (solid lines) and *p* > .05 (dashed lines), standard errors (bars), and confidence intervals (shaded) are shown

Pied flycatchers experienced an overall advancement of annual‐average hatching time across Sweden during the study period. In the northern latitudinal band, this change was 8.3 days earlier (0.23 days/year; *N* = 26 years, *b* (slope) = −0.23, *SE* = 0.05, *t*_24_ = −4.94, adjusted *R* ^2^ = 0.48, *p* < .001; Figure 5a). In the southern band, the change was 3.6 days earlier (0.1 days/year; *b* = −0.09, *SE* = 0.04, *t*_24_ = −2.07, adjusted *R* ^2^ = 0.12, *p* = .049; Figure 5a). In the intermediate band, the average showed the same magnitude of change as in the southern band (3.6 days earlier) although this trend was near significant (*b* = −0.09, *SE* = 0.05, *t*_24_ = −1.94, adjusted *R* ^2^ = 0.10, *p* = .06; Figure 5a). In accordance with this, the model averaging showed a high relative importance of year (*w_i_* = 1) at the three latitudinal bands indicating earlier breeding during the study period, but the year effect was weaker at the northern band (Table 2). Furthermore, the model averaging showed a high relative importance of prehatching temperature at all latitudinal bands indicating earlier breeding with higher temperatures and with the strongest effect at the northern band (Table 2; estimates from −0.28 to −0.43; Figure 6a). The interaction between temperature and year had high relative importance at all latitudinal bands, with positive estimates indicating that the effect of the temperature depended on the year and its effect diminished lightly over time (Table 2).

NDVI had high relative importance at all latitudinal bands, with more greenness rendering earlier hatching time, and the effect was strongest at the northern band (Table 2 and Figure 6b). At the southern and intermediate bands, specifically, the interaction between NDVI and year showed a relatively important effect with negative low estimates, indicating that the effect of NDVI depended on the year and the effect of higher NDVI on earlier breeding time was slightly stronger over time. However, the interaction of NDVI and year was not important at the northern band. Rain and distance to the coast had effects only at the intermediate latitudinal band, with positive estimates indicating that the more rain and inland (especially at the most inland level compared with the others), the later hatching (Table 2; but see Figure 6c where we observed no association). Latitude, as continuous variable, had a relatively high effect showing later hatching to the north up to the intermediate latitudinal band. However, at the northern band, its effect was negative (Table 2).

### 3.3 Effects on breeding success

**FIGURE 7:**
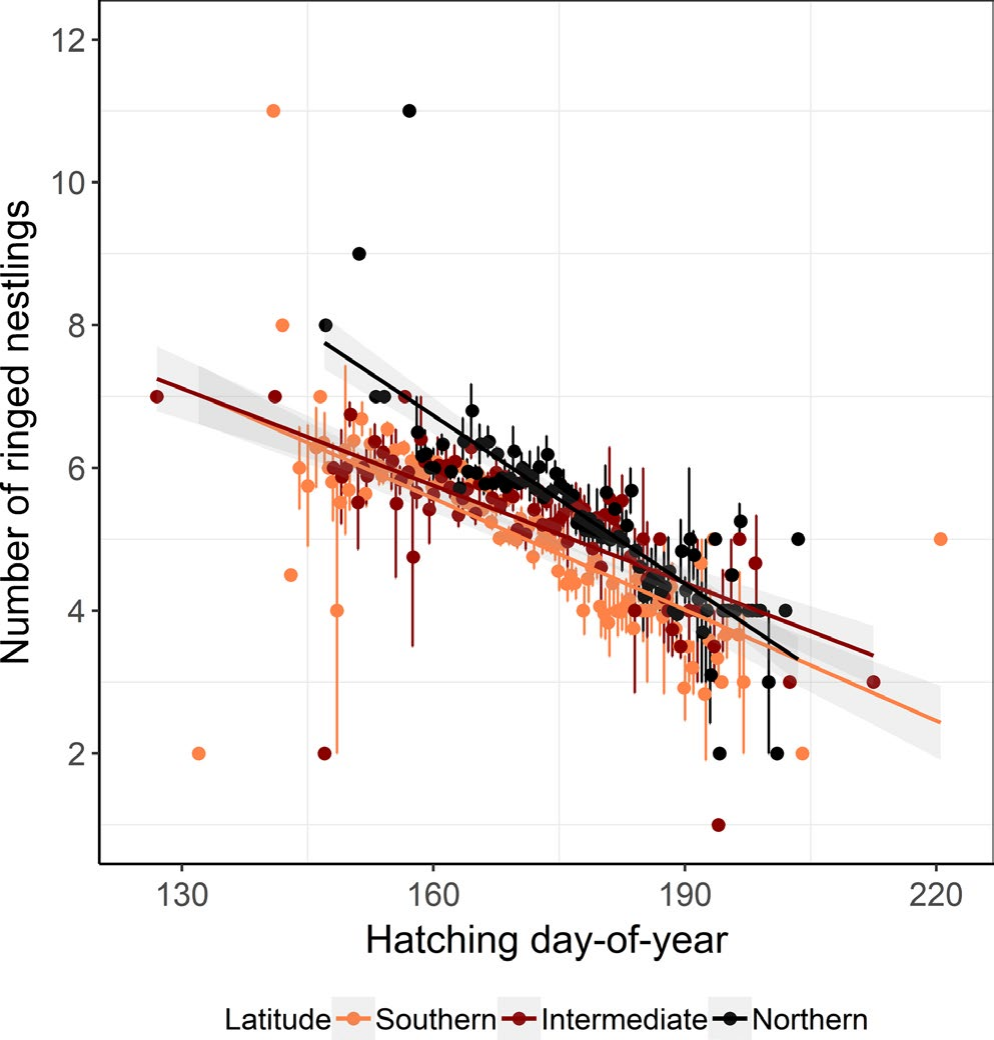
Breeding success in relation to breeding time within season. Average number of ringed nestlings per hatching day‐of‐year for pied flycatchers breeding in Sweden from 1982–2017 split by three latitudinal bands. Linear regression of ringed nestlings ~ hatching day‐of‐year: southern, *b* (slope) = −0.05, *SE* = 0.005, *t*_108_ = −10.55, adjusted *R* ^2^ = 0.50, *p* < .001; intermediate, *b* = −0.04, *SE* = 0.005, *t*_90_ = −9.39, adjusted *R* ^2^ = 0.49, *p* < .001, and northern, *b* = −0.08, *SE* = 0.005, *t*_86_ = −14.32, adjusted *R* ^2^ = 0.70, *p* < .001. Linear regressions with *p* ≤ .05 (solid lines), standard errors (bars), and confidence intervals (shaded) are shown

One of the most important variables associated with breeding success across all latitudinal bands was hatching day‐of‐year, indicating that earlier breeding birds had higher reproductive success (Table 3 and Figure 7). At the northern band, pied flycatchers increased annual‐average breeding success throughout the study period with +0.01 chicks/year, while in the intermediate and southern bands, there were no directional changes over time (northern, *b* = 0.01, *SE* = 0.005, *t*_24_ = 2.78, adjusted *R* ^2^ = 0.21, *p* = .01; intermediate, *b* = −0.01, *SE* = 0.005, *t*_24_ = −1.17, adjusted *R* ^2^ = 0.01, *p* = .25, and southern, *b* = 0.005, *SE* = 0.003, *t*_24_ = 1.69, adjusted *R* ^2^ = 0.07, *p* = .10; Figure 5b).

At the southern and intermediate latitudinal bands higher prehatching temperature had a negative influence on breeding success (Table 3). In addition, at the southern band we found a relatively high effect of latitude suggesting an increase in success toward the north (Table 3). Finally, across all latitudinal bands, the younger the chicks were when ringed, the higher breeding success was found (more chicks were alive per nest; Table 3; chick age).

## 4 DISCUSSION

### 4.1 Climate change over the study period

Overall, our analyses of temperature and vegetation greenness during 1982–2017 are in accordance with reported temperature increase and earlier vegetation period in Sweden during the last decades (SMHI, 2021b, 2021d). During the prehatching period, vegetation greenness greatly increased over the years at all sampled locations, while an increase in temperature was observed only in the southern latitudinal band. No change in precipitation was found at the sampled locations, which is in line with the small precipitation changes reported for Sweden from 1980 (SMHI, 2021c). Although the temperature increase found during the prehatching period is lower than expected at high latitudes (Root et al., 2003; SMHI, 2021b, 2021d), earlier vegetation development over the years suggests overall warmer conditions in the north as well. Cumulative effects of warmer temperature and precipitation during earlier or longer periods of the year than measured in this study may have influenced the more intense change observed in vegetation greenness (Schwartz et al., 2006).

### 4.2 Effects on breeding time

Our study shows that pied flycatchers breeding in Sweden advanced their breeding time during a period of 36 years. The change was largest in the northern part of Sweden, where hatching day advanced on average 8.3 days, compared with southern Sweden where the advance was on average 3.6 days over the study period. Our observed large advancement in breeding time in the north of Sweden suggests that migration timing and migration distance did not markedly constrain pied flycatchers from being flexible to an earlier spring at their northernmost distribution range.

The most important factors for the advanced breeding were the increasing temperatures and vegetation greenness index in May all over Sweden and also in the first half of June at northernmost latitudes. A relatively stronger effect of temperature and greenness on breeding time in the north suggests that pied flycatchers may be more responsive to weather cues at higher latitudes, as they face more stochastic weather conditions and are more time constrained due to shorter breeding seasons (Conklin et al., 2010; Järvinen & Väisänen, 1984). Our results are in line with a long‐term study in North America where it was shown that purple martins *Progne subis* lay eggs earlier in warmer springs and this effect was stronger toward the north (Shave et al., 2019). The fact that there is less human activity influencing the dynamics of vegetation greenness in the north of Sweden may make the greenness index a better measurement of spring phenology in the north compared with more southern latitudes (Pettorelli et al., 2005). Our observation of advanced breeding time with increasing temperature in northern Sweden contrasts with previous long‐term studies of pied flycatchers in the northern distribution range that found stable (Kluen et al., 2017; Nyholm, 2011; Ram et al., 2019) or later (Both et al., 2004; Laaksonen et al., 2006) breeding time over the years. Different rates of climate change depending on latitude and study period (Both & Marvelde, 2007; Visser et al., 2015) as well as the geographical scale under study (Laaksonen et al., 2006; Ram et al., 2019) may influence the response found on breeding time. Compared with the previous studies, we sampled more scattered locations over Sweden, thus maybe representing a larger variation of the pied flycatcher breeding situation at the northern breeding range. Our findings for southern Sweden are, however, supported by studies of more southerly populations of pied flycatchers that showed earlier spring migration and egg laying in connection with temperature increase (Both et al., 2004; Cadahía et al., 2017; Hüppop & Winkel, 2006).

The effect of prehatching temperature on breeding time turned out to weaken over the years indicating that temperature became less important for breeding time. At the same time, in the intermediate and southern latitudinal bands, vegetation greenness had a successively stronger long‐term effect on hatching time. This highlights the importance of vegetation greenness, influencing breeding time throughout the study period and, potentially, a delayed effect of temperature increase in the long term. The fact that we got a somewhat unexpected result in the northernmost area with earlier breeding time with higher latitude could be related to the topography and influence of the sea—and thus climate conditions at the sampled areas—since many nests in the very north were located at the coast (Figure 2).

At intermediate latitudes, more precipitation and longer distance to the coast were associated with delayed breeding. In this area, at approximately 60°N, we find the bioclimatic transition zone ‘Limes Norrlandicus’ separating the warmer southern mixed forest from the colder northern boreal forest (Gullefors, 2008), where precipitation increases from coast to inland (Greiser et al., 2020; SMHI, 2021a). We can thus expect that pied flycatchers breeding in this area face more variable habitat in terms of vegetation and climate than at other latitudes due to the transition zone.

### 4.3 Effects on breeding success

Birds breeding early in the year may have larger clutches and longer seasons to rear their young (Dunn, 2004; Shave et al., 2019; Tomotani et al., 2018), and specifically, early breeding pied flycatchers may lay more eggs (Järvinen, 1989), giving opportunity for a higher reproductive success compared with later breeding birds. Accordingly, our results show that pied flycatchers that bred early in the season had more chicks in the nest at ringing than later breeders. Furthermore, number of ringed chicks in the nest, our measurement of breeding success, increased in the north throughout the study period while it did not change at more southern latitudes. Our results with stronger response of breeding time advancement in pied flycatchers at northernmost Sweden may have contributed to the observed increase in breeding success. Northern pied flycatchers did on average increase breeding success with 0.36 chick during the study period of 36 years, indicating that they nowadays perform as the more southerly populations under study or maybe even better. Perhaps, reproductive success of northernmost populations of pied flycatchers may be favored by higher temperatures as suggested for songbirds breeding in boreal areas in Finland (Meller et al., 2018). At subarctic areas, harsher climate conditions and a more generalist bird community (Eeva et al., 2000) might result in lower competition for territories and food (Herrera, 1978). This may have contributed to such increase in breeding success in the north, enhanced by the recent milder climate.

Breeding earlier may, however, not always correspond to higher breeding success as other factors like trophic mismatch, extreme weather events (Dunn, 2004; Visser et al., 1998, 2015), competition at high breeding densities (Ahola et al., 2012), and more frequent competitive interactions (Samplonius & Both, 2019) may render lower reproductive success. At southern and mid‐latitudes, we found no change in breeding success, despite earlier breeding time in the south. Interestingly, at these latitudes, in accordance with a study of a more southern population of pied flycatchers (González‐Braojos et al., 2017), high prehatching temperatures rendered a reduced breeding success. This result suggests that in years with early and warm spring conditions, even early breeding flycatchers might be too late to time the peak of food availability for their chicks (Both et al., 2006). Furthermore, especially during years with warm winters and early springs, it has been shown that resident and short‐distance migrants can have an advantage over long‐distance migrants when competing for good quality territories (Herrera, 1978). This could thus negatively influence breeding success of pied flycatchers if they have to occupy lower quality territories, as suggested in studies of interactions between tits and flycatchers (Ahola et al., 2007; Samplonius et al., 2018; Samplonius & Both, 2019).

In summary, our study indicates that a long‐distance migratory songbird breeding at the north of its range is able to adapt breeding time to climate change by tracking the earlier vegetation greenness over the years despite the long distance between breeding and wintering grounds. Furthermore, birds breeding in the very north of Sweden showed an increase in breeding success and the largest advancement in breeding time over the study period, indicating that migration time schedule does not constrain the breeding time response to spring advancement. However, in southern and mid‐latitudes of Sweden, negative effects of warm spring temperatures on breeding success suggest an inability to fully adjust breeding time to the prevailing spring conditions at such occasions. The observed stronger response of breeding time and breeding success in the north may relate to a tighter adjustment of breeding time to the environment due to shorter breeding seasons than birds breeding at more southern latitudes in Sweden.

Even if some long‐distance migrants can adjust their breeding time to climate change so far and evolutionary changes in spring timing have been shown in pied flycatcher (Helm et al., 2019), a critical question is whether they can breed even earlier in the future. On the basis of rapid global warming projected in the future (Diffenbaugh & Field, 2013), with stronger effects toward higher latitudes in the Northern Hemisphere (IPCC, 2001; Parmesan, 2007; Shukla et al., 2019), there is a high risk that long‐distance migrants, especially in their northernmost breeding range, at some point will be unable to fully adjust breeding time in response to climate change. The reduced breeding success in Swedish pied flycatchers at southern and mid‐latitudes during warm springs reported in our study could thus be expected to also occur in more northern breeding birds with continued climate change and indicates a fate that could be true for several long‐distance migratory species (Radchuk et al., 2019; Visser, 2019).

## CONFLICT OF INTEREST

The authors declare no conflict of interests.

## AUTHOR CONTRIBUTIONS

**Marta Lomas Vega:** Conceptualization (equal); data curation (lead); formal analysis (lead); funding acquisition (supporting); investigation (lead); methodology (lead); project administration (equal); resources (equal); software (lead); validation (lead); visualization (lead); writing‐original draft (lead); writing‐review & editing (lead). **Thord Fransson:** Conceptualization (lead); data curation (lead); funding acquisition (lead); investigation (supporting); methodology (supporting); project administration (equal); resources (equal); supervision (lead); validation (supporting); visualization (supporting); writing‐original draft (supporting); writing‐review & editing (equal). **Cecilia Kullberg:** Conceptualization (lead); data curation (supporting); funding acquisition (lead); investigation (supporting); methodology (supporting); project administration (equal); resources (equal); supervision (lead); validation (supporting); visualization (supporting); writing‐original draft (supporting); writing‐review & editing (equal).

## Supporting information

Table S1‐S2

## Data Availability

The data supporting the study are available at the Dryad Digital Repository https://doi.org/10.5061/dryad.v9s4mw6v4.
