# Supplementary material for: The effects of four decades of climate change on the breeding ecology of an avian sentinel species across a 1,500‐km latitudinal gradient are stronger at high latitudes
Source: Ecol Evol. 2021 Mar 18;11(11):6233–47. doi: 10.1002/ece3.7459 (PMC8207436; doi:10.1002/ece3.7459)
Supplement: Supplementary file 1 — Table S1‐S2 [file ECE3-11-6233-s001.docx]

# Appendix

**Table S1.** **Characteristics of competing linear mixed-effect models explaining the variation of breeding time in function of the environment**. Hatching day-of-year was used as response variable and location ID as a random factor in all models. Fixed effects belong to the three top-competing models per latitudinal band and they were obtained through model averaging. Models were ranked by AIC, with best-supported models having the lowest AIC or it differed ≤ 2 units from the top-competing model. The second-order AIC (AICc) was not used since N/K > 40 (N = number of observations, K = number of model parameters; Burnham & Anderson, 2002). ΔAIC shows the difference between AIC of a given model and the top-competing model. Akaike weights (*w_i_*), number of model parameters (K) and time windows analysed are shown. Time window refers to the environment analysed for the given periods, with reference to mean hatching periods. A, B & C refer to 15-day periods before mean hatching period as described in Figure 3 (of main text). Mean hatching period was first half of June at southern and intermediate latitudinal bands while it was second half of June at the northern band. Null models had the intercept and the random factor as model terms.

|  | Fixed effects | ΔAIC | *w_i_* | K | Time window  (periods as in Fig. 3) |  |
| --- | --- | --- | --- | --- | --- | --- |
| Southern latitudinal band | Year*Temperature + Year*NDVI + Latitude | 0.00 | 0.88 | 9 | May 1 - 31 (A & B) |  |
|  | Year*Temperature + Year*NDVI + Latitude + Distance to the coast | 4.17 | 0.11 | 10 | May 1 - 31 (A & B) |  |
|  | Year*Temperature + Year*NDVI + Precipitation + Latitude | 9.98 | 0.01 | 10 | May 1 - 31 (A & B) |  |
|  | Null model | 1450.84 | 0.00 | 3 |  |  |
| Intermediate latitudinal band | Year*Temperature + Year*NDVI + Precipitation + Latitude + Distance to the coast | 0.00 | 0.76 | 12 | May 1 - 31 (A & B) |  |
|  | Temperature + Year*NDVI + Precipitation + Latitude + Distance to the coast | 4.31 | 0.09 | 11 | May 1 - 31 (A & B) |  |
|  | Year*Temperature + Year*NDVI +Year*Precipitation + Latitude + Distance to the coast | 5.53 | 0.05 | 13 | May 1 - 31 (A & B) |  |
|  | Null model | 554.21 | 0.00 | 3 |  |  |
| Northern latitudinal band | Year*Temperature + NDVI + Latitude | 0.00 | 0.55 | 8 | May 1 - June 14 (A, B & C) | |
|  | Year*Temperature + NDVI + Latitude + Distance to the coast | 2.28 | 0.17 | 10 | May 1 - June 14 (A, B & C) | |
|  | Year*Temperature + NDVI + Year*Precipitation + Latitude | 3.24 | 0.11 | 10 | May 1 - June 14 (A, B & C) | |
|  | Null model | 551.95 | 0.00 | 3 |  |  |

**Table S2.** **Characteristics of competing linear mixed-effect models explaining the variation of breeding success in function of the environment**. Number of ringed chicks was used as response variable and location ID as a random factor in all models. Fixed effects belong to the three top-competing models per latitudinal band and they were obtained through model averaging. Models were ranked by AIC, with best-supported models having the lowest AIC or it differed ≤ 2 units from the top-competing model. The second-order AIC (AICc) was not used since N/K > 40 (N = number of observations, K = number of model parameters; Burnham & Anderson, 2002). ΔAIC shows the difference between AIC of a given model and the top-competing model. Akaike weights (*w_i_*), number of model parameters (K) and time windows analysed are shown. Time window refers to the environment analysed for the given periods, with reference to mean hatching periods. A & B refer to 15-day periods before mean hatching period as described in Figure 3 (of main text). Mean hatching period was first half of June at southern and intermediate latitudinal bands while it was second half of June at the northern band. “Hatch.day” is hatching day-of-year. Null models had the intercept and the random factor as model terms.

|  | Fixed effects | ΔAIC | *w_i_* | K | Time window (periods as in Fig. 3) |
| --- | --- | --- | --- | --- | --- |
| Southern latitudinal band | Hatch.day + Chick age + Temperature + Latitude | 0.00 | 0.56 | 7 | May 1 - 31 (A & B) |
|  | Hatch.day + Chick age + Temperature + Latitude + Year | 2.88 | 0.13 | 8 | May 1 - 31 (A & B) |
|  | Hatch.day + Chick age + Temperature+ Latitude + Distance to the coast | 3.83 | 0.08 | 8 | May 1 - 31 (A & B) |
|  | Null model | 1474.49 | 0.00 | 3 |  |
| Intermediate latitudinal band | Hatch.day + Chick age + Temperature | 0.00 | 0.75 | 6 | May 1 - 31 (A & B) |
|  | Hatch.day + Chick age + Temperature + Year | 3.73 | 0.12 | 7 | May 1 - 31 (A & B) |
|  | Hatch.day + Chick age + Temperature + Latitude | 5.70 | 0.04 | 7 | May 1 - 31 (A & B) |
|  | Null model | 207.45 | 0.00 | 3 |  |
| Northern latitudinal band | Hatch.day + Chick age | 0.00 | 0.67 | 5 |  |
|  | Hatch.day + Chick age + Latitude | 4.35 | 0.08 | 6 |  |
|  | Hatch.day + Chick age + Distance to the coast | 5.42 | 0.04 | 7 |  |
|  | Null model | 403.81 | 0.00 | 3 |  |

# Appendix references

Burnham, K. P., & Anderson, D. R. (2002). *Model selection and multimodel inference: a practical information-theoretic approach.* (2nd ed.). New York: Springer.
